# Effectiveness of Silica Coatings in Enhancing Resin Cement Adhesion to Zirconia: A Systematic Review

**DOI:** 10.3390/dj13090426

**Published:** 2025-09-15

**Authors:** Laura C. Lara-Hernández, Luis C. Jiménez-Borrego, Nelly S. Roa

**Affiliations:** 1 Centro de Investigaciones Odontológicas (CIO), Departamento del Sistema Dentario, Facultad de Odontología, Pontificia Universidad Javeriana, Bogotá 110231, Colombia; 2 Grupo de Películas Delgadas y Nanofotónica, Departamento de Física, Facultad de Ciencias, Pontificia Universidad Javeriana, Bogotá 110231, Colombia; cjimenez@javeriana.edu.co; 3 Centro de Investigaciones Odontológicas (CIO), Departamento del Sistema Bucal, Facultad de Odontología, Pontificia Universidad Javeriana, Bogotá 110231, Colombia

**Keywords:** adhesion, resin cements, shear strength, silicones, surface properties, zirconium

## Abstract

**Background:** The use of zirconia-based restorations has increased in dentistry due to their aesthetics, high strength, and biocompatibility. However, achieving durable adhesion between resin cements and zirconia remains a challenge in restorative dentistry. Adhesive failures can lead to complications ranging from dentin hypersensitivity to the loss of the restored tooth. This review evaluates the impact of surface treatments based on silica coatings as a strategy to improve the adhesion of resin cements to zirconia. **Methods**: A systematic review was conducted following PRISMA guidelines. Searches were performed in Scopus, Web of Science, PubMed, EBSCO, and ScienceDirect. In vitro studies were included if they (1) evaluated silica-based coatings on zirconia surfaces; (2) measured bond strength at the zirconia–cement interface through shear tests (MPa) and reported failure type (adhesive, cohesive, mixed); (3) used ≥8 specimens per group; and (4) included an untreated zirconia control group. Data were extracted and compared for conditions before and after thermocycling in the studies that reported this procedure. **Results**: The average bond strength for silica-coated zirconia was 15 MPa without thermocycling and 11.97 MPa after thermocycling, regardless of the coating technique. These values were significantly higher than those of untreated zirconia (8.45 MPa and 6.41 MPa, respectively). Cohesive and mixed failures predominated in silica-treated groups, suggesting more effective adhesion compared to controls, which presented mainly adhesive failures. **Conclusions**: Silica coatings, especially when combined with adhesion promoters, MDP-based primers, significantly enhance the bond strength of resin cements to zirconia. This reduces the risk of secondary caries, sensitivity, restoration debonding, and potential tooth fractures. However, the effectiveness of these coatings varies depending on the technique used, suggesting the need to standardize protocols to optimize clinical outcomes.

## 1. Introduction

In recent decades, metal-free restorations have become the materials of choice for both dentists and patients. This is primarily due to the benefits that metal-free materials offer in terms of aesthetics, functionality, and biocompatibility. The high demand for replacement and/or changes in restorative treatments, mainly in the adult population, in procedures performed by general dentists is approximately 50% [1]. Additionally, the need for restorative treatments with crowns increases with age because older adults present higher prevalence of dental structure loss, wear, and previous treatments that increase the indication for treatment with restorations such as crowns [2]. Primarily aesthetic and biological reasons have led to a significant increase in demand and use of metal-free materials such as zirconia crowns (tetragonal phase stabilized with 3 mol% yttrium, 3Y-TZP) [3,4]. Their clinical applications are very broad, but they are especially found in posterior prostheses where there is a high occlusal load and masticatory impact [5]. The mechanical and optical properties of zirconia are defined by the yttrium content that stabilizes the phase. 3Y-TZP zirconia has greater fracture toughness and flexural strength compared to zirconia with higher yttrium content such as zirconia stabilized with 4 mol%, 5 mol%, and 6 mol% [6,7]. Although zirconia is known for having advantages such as being an inert, opaque, and highly biocompatible material, it presents an important disadvantage regarding adhesion with resin cements used to bond zirconia to dental tissue; achieving adequate adhesion of these cements to zirconia is a challenge [8,9].

In the process of bonding zirconia to dental tissue through resin cement, two interfaces are created: cement–zirconia and cement–dental tissue. The cement–dental tissue bond interface is highly studied, and a more predictable bond is recognized than the bond at the zirconia–cement interface. The difficulty in adhesion at the zirconia–cement interface is due to the low-reactive surface of zirconia; it is not etchable with acids and presents high hardness and crystallinity that make zirconia insensitive to conventional adhesion protocols [10,11,12,13,14]. These characteristics of the zirconia surface make it a hydrophobic material, with a contact angle between 70° and 85° that hinders surface wettability by the cement [13,14,15]. The adhesion of cement to restorations is not only influenced by factors of the restorative material surface but also depends largely on the choice of cement agent, the cementation process, and achieving intimate contact between the cement and zirconia. In this case, the zirconia–cement contact is compromised, resulting in poor bonding at the zirconia–cement interface. For this reason, zirconia restorations tend to have inadequate marginal adaptation, presenting lower crown retention to the dental substrate and generating premature failures.

To address the problem of resin cement adhesion to the zirconia surface, various surface treatments have been proposed: chemical, physical, mechanical, or combinations of several treatments. Some physical/mechanical treatments include alumina sandblasting, laser irradiation, ceramic coating, glass infiltration, and tribochemical silica coating (TBS) including tribochemical methods such as CoJet and Rocatec, widely used in clinical practice [11,16]. In recent years, coating the zirconia surface with SiO_2_ films through mainly physicochemical techniques has been explored with special interest [15,16]. Some of the physicochemical techniques employed are atomic layer deposition (ALD), sol–gel technique, dip-coating techniques, and vapor deposition techniques including magnetron-assisted and radio frequency sputtering [17,18,19].

To adequately understand SiO_2_ surface treatments, it is important to delve into the characteristics of each technique mentioned above. Tribochemical silica coating involves the high-pressure application of silica-coated alumina particles onto the zirconia surface, creating a reactive silica layer that enhances chemical bonding with silane-based primers and resin cements. This method is widely used in clinical dentistry due to its simplicity and effectiveness [20]. Atomic layer deposition (ALD) allows precise control of SiO_2_ film thickness at the nanometric level (10–100 nm), generating uniform, dense, and highly adherent coatings through sequential chemical reactions in the gas phase [17,21]. The sol–gel technique, more economical and accessible in clinical environments, allows us to obtain layers of 100–500 nm through hydrolysis and condensation of liquid precursors, although it presents greater variability in terms of thickness and homogeneity [22]. Dip-coating techniques offer simplicity but less thickness control, varying between 50 and 300 nm [23,24]. Sputtering generates thinner layers (30−80 nm) with excellent adherence due to high-energy ionic bombardment that creates a gradual transition interface [25], in this case between zirconia and SiO_2_.

Each technique presents advantages and limitations. Atomic layer deposition (ALD) offers superior thickness control and conformality and the ability to create uniform coating on a three-dimensional surface including side walls, corners, cavities, and structures with a high aspect ratio, maintaining constant thickness throughout the surface [21,26]. The disadvantage with this technique is that it requires expensive equipment and prolonged processing times, limiting its clinical applicability. Sol–gel and dip-coating techniques stand out for their accessibility and ease of implementation in dental environments, although they present greater variability in results. Sputtering techniques generate excellent adherence but require specialized vacuum equipment. These technical differences are directly reflected in the quality of the adhesive interface and bond strength values, with studies reporting adhesion increases from 20% to 150% compared to untreated zirconia, depending on the technique used and its specific processing parameters [18,23,27].

Many dentists commonly use dual-cure self-adhesive resin cements (SARCs) to cement zirconia restorations because they combine the advantages of adhesive agents and conventional cements in a single-step system that has demonstrated better adhesion to zirconia compared to total-etch systems [28]. Consequently, the clinical process is simplified, reducing possible errors and contamination [29,30]. SARCs used in zirconia cementation present a complex chemical composition designed to achieve adhesion without additional steps. These cements generally contain (i) an organic matrix composed of methacrylate monomers such as Bis-GMA, UDMA, or TEGDMA; (ii) functional acidic monomers with phosphate or carboxylic groups that provide initial acidity and chemical adhesion capacity; (iii) silanized inorganic fillers to mechanically reinforce the cement (barium glasses, colloidal silica, fluorides); (iv) dual initiator systems that combine photoinitiators and chemical initiators to ensure adequate polymerization in areas without light access; and (v) stabilizing agents, inhibitors, and rheological controllers [30,31].

The adhesion mechanism of SARCs differs between surfaces with and without silica content. On silica-containing surfaces, the initial acidity of the resin cement allows micromechanical retention by having some interaction with surface irregularities [30]. In addition to these retentions, covalent bonds (Si-O-Si) are formed between silanol groups of the silanized surface and silane groups found in the cement [32,33]. Furthermore, some secondary interactions (Van der Waals forces and hydrogen bonds) can form and complement the primary bond that has already been formed [28,33]. In contrast, on silica-free surfaces (as occurs in untreated zirconia), adhesion is mainly limited to reduced micromechanical retention due to zirconia’s high crystallinity and interaction between phosphate groups with zirconia metal oxides, forming bonds (Zr-O-P) that present lower hydrolytic stability [34,35].

Among the most clinically used SARCs, differences are observed in their composition both in fillers and organic matrices. These compositional changes can influence experimental results because they generate differences in initial pH, viscosity, working time, and film thickness [36], factors that directly affect adhesive quality and should be considered when interpreting comparative results between studies. It is important to understand that most dentists use silane-based treatments for cementation with resin cements, which highlights the importance of developing treatments compatible with silane chemistry in materials like zirconia. However, due to its lack of silica content, the zirconia surface does not respond to common chemical modifications such as hydrofluoric acid etching or silanization [19,37]. The use of aggressive mechanical treatments on the zirconia surface can cause premature catastrophic failures, changes in the tetragonal phase, and the appearance of cracks and fissures [38]. Therefore, it becomes crucial to understand how modifications in the zirconia surface structure with silica atoms could potentially improve adhesion techniques without modifying the bulk material properties.

The chemical treatment used as a complement to these surface treatments, both mechanical and physicochemical, employs different substances as adhesion promoters: primers containing the 10-methacryloyloxydecyl dihydrogen phosphate (MDP) molecule, functional primers, and silanes to increase surface energy and cement wettability [39]. These chemical substances improve adhesion between the organic phase of the resin cement and the inorganic phase of zirconia [37,38,39]. Silica surface treatments have shown potential improvements in SARC adhesion to zirconia [40], especially in conjunction with the use of chemical adhesion promoters, but variability in research results represents an important challenge. Despite different methodologies for evaluating bonding at the zirconia-cement interface, shear stress tests on the bonding interface remain the most employed laboratory tests, standardized by ISO 29022:2013 [41].

The ISO 29022:2013 standard provides important information for evaluating bond strength at the zirconia–cement adhesive interface. It indicates the need for very rigorous methodological control to obtain reproducible and clinically relevant results. Shear stress tests require precise alignment devices that guarantee perpendicularity between the treated surface and the force application direction [41]. In these in vitro tests, untreated zirconia presents bond strength values around 4 MPa and low values when compared to other treatments such as aluminum oxide sandblasting and SiO_2_ coatings, which present values between 8 MPa and 13 MPa [17,42]. This increase in adhesion values to the zirconia surface demonstrates the importance of performing zirconia surface conditioning prior to cementation.

In addition to bond strength at the interface under shear stress, adhesion durability to zirconia represents a critical challenge in clinical conditions. Thermocycling is a widely accepted artificial aging method that simulates thermal changes in the oral cavity and allows the determination of the long-term behavior of the achieved adhesion [35]. Thermocycling consists of subjecting samples to repeated cycles of extreme temperatures (generally between 5 °C and 55 °C) for thousands of cycles. This technique, standardized in ISO 11405:2015 [43], allows the evaluation of hydrolytic degradation of the adhesive interface, which is especially relevant for Zr-O-P bonds formed between zirconia and some cements that present greater susceptibility to hydrolysis compared to Si-O-Si bonds. Studies such as those by Ozcan and Bernasconi [35] and Erdem et al. [44] have demonstrated significant reductions in adhesive strength post-thermocycling, with decreases ranging between 20% and 50% depending on the applied surface treatment. The surfaces least affected by thermocycling were silica-treated surfaces when used in conjunction with appropriate silane coupling agents, as reported by Rigos et al. [45] and Mantinlinna et al. [34].

In analyzing bond strength between dental materials, evaluation of the failure type occurring after mechanical testing is fundamental for interpreting the quality of achieved adhesion. This failure is defined as the rupture that occurs at the interface between two materials [43,46]. Some standards that standardize these types of failures are ISO 29022:2013, ISO 11405:2015, ISO 4049:2019, and ISO 10365:2022 [41,43,46,47]. These standards recommend documenting the failure type to provide quality and efficacy of bonding between materials [47]. The standardized methodology for this evaluation combines complementary microscopic techniques. Generally, observation begins with stereoscopic optical microscopy to identify general distribution of failure patterns, and subsequently, if possible, scanning electron microscopy observation should be performed to characterize microstructural details of interfaces.

Considering the ISO standards mentioned above and carrying out adequate microscopic observation, these failures can be classified as follows: (1) adhesive failures—when separation occurs at the interface between two materials of different nature, in this case resin cement and zirconia surface, should not present visible cement residues on the zirconia surface. (2) Cohesive failures—when rupture occurs within one of the involved materials (cement or restorative material) and structural continuity is maintained at interfaces. Cohesive failures are an indication that the bond is strong and that fracture occurs within the material and not at the adhesive interface, suggesting good adhesion. (3) Mixed failures—when a combination of adhesive and cohesive failure is observed. Mixed failures reflect an intermediate situation, indicating partial problems in adhesion that merit review [47,48,49].

The prevalence of cohesive or mixed failures is usually interpreted as an indication of stronger and more effective bonding, while a high proportion of adhesive failures may indicate weakness in interfacial interaction. Although it is not quantitatively stipulated how to determine if it is one type of failure or another, some authors consider that if a single type of failure is present between 70% and 80% of the surface, it will be classified as that type of failure [48,49]. Quantification of the percentage of each type of failure can be performed using image analysis software.

For this reason, the objective of this study is to determine, through a systematic review of available literature, whether zirconia surface treatments with SiO_2_ increase shear bond strength of SARCs compared to conventional treatments. Our hypothesis is that SiO_2_ coatings not only increase bond strength values in megapascals (MPa) but also favorably modify the failure pattern, increasing the proportion of cohesive and mixed failures, which would indicate substantial improvement in the quality of the zirconia–cement adhesive interface.

## 2. Materials and Methods

The aim of this review is to critically evaluate the available scientific evidence regarding the effectiveness of silica-based surface treatments in enhancing the adhesion of dual-cure self-adhesive resin cements (SARCs) to zirconia. Given the growing clinical use of zirconia restorations and the persistent challenges in achieving durable bonding, a systematic synthesis of in vitro studies was deemed necessary to identify trends, compare treatment protocols, and highlight areas requiring further research.

The research question was structured following the PICOT framework: population (P)—in vitro studies evaluating yttria-stabilized zirconia; intervention (I)—application of silica-based surface coatings; comparison (C)—untreated zirconia surfaces; outcomes (O)—bond strength values (MPa) and type of failure (adhesive, cohesive, mixed); time (T)—before and after thermocycling, when this procedure was reported or performed in the included studies.

For the development of this review, a structured search strategy was followed in Scopus, Web of Science, PubMed, EBSCO, and ScienceDirect databases using combinations of keywords and MESH terms such as Zirconia, yttria stabilized zirconia, 3Y-TZP, Surface treatment, thin film, coating, Surface conditioning, Silicon oxide, Silica, silicon dioxide, Resin cement, dual cure resin cement, luting cement, bond strength and bond strength test. Using Boolean operators, an advanced search was initiated at least for the title, abstract, and keywords with the following base algorithm: (TITLE-ABS-KEY (zirconi*) OR TITLE-ABS-KEY (“yttria stabilized zirconia”) OR TITLE-ABS-KEY (“3y tzp”) AND TITLE-ABS-KEY ( “surface treatment*”) OR TITLE-ABS-KEY (“thin film*”) OR TITLE-ABS-KEY (coating*) OR TITLE-ABS-KEY (“surface conditioning”) AND TITLE-ABS-KEY (“silicon dioxide”) OR TITLE-ABS-KEY (silica) AND TITLE-ABS-KEY (“resin cement*”) OR TITLE-ABS-KEY (“dual cure resin cement”) OR TITLE-ABS-KEY (“luting cement*”) AND TITLE-ABS-KEY (“bond strength”) OR TITLE-ABS-KEY (“bond strength test”) AND NOT TITLE-ABS-KEY (implant)). Based on this algorithm, modifications were made for different databases (Appendix A: Table A1).

In each of the databases used in this review, automatic alerts were configured to maintain weekly literature updates. The literature search was not restricted in time to obtain the greatest amount of information possible. The last search was conducted on 18 May 2025.

**Figure 1:**
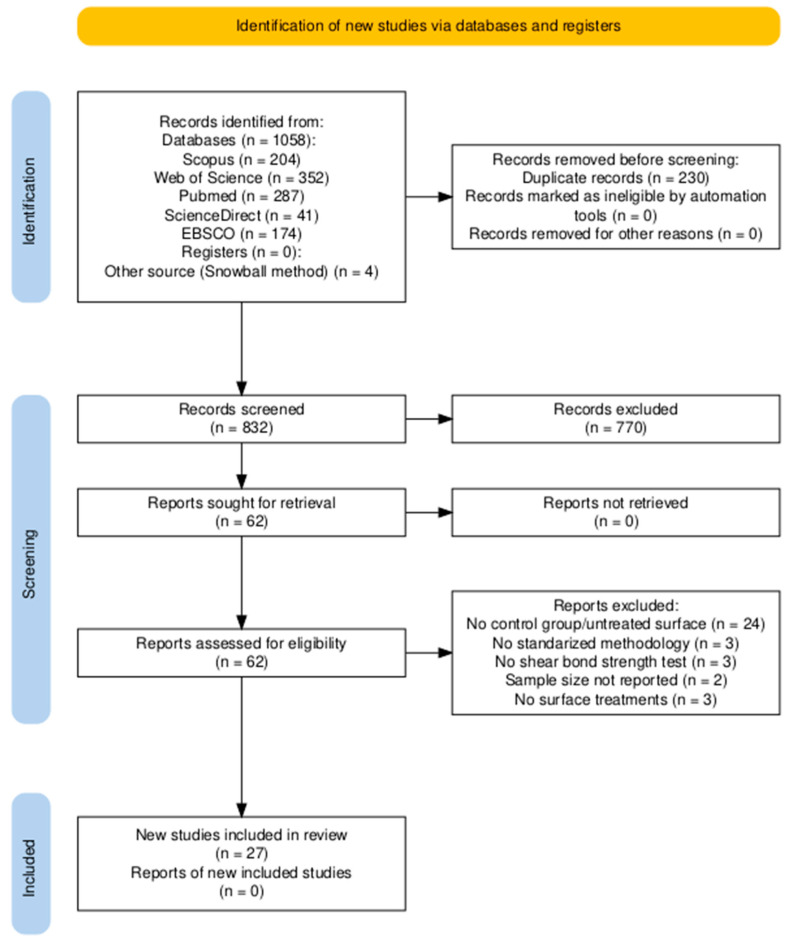
PRISMA diagram with the search methodology and study selection. Includes consulted databases, exclusion criteria and the number of records identified, filtered, evaluated, and finally included in the review.

Information obtained from each database was exported in RIS format to Mendeley, where a library was created for this systematic review. Duplicate references were identified and removed using Mendeley software. Titles and abstracts of investigations were independently screened by two reviewers. Inclusion criteria (Figure 1) were (1) publications of quantitative experimental in vitro studies that evaluated bond strength of resin cements on zirconia surfaces; (2) investigations that included silica as a zirconia surface treatment in some of their experimental groups (TBS, sputtering processes, sol–gel, ALD, and dip coating); (3) assays that examined bond strength through shear tests; (4) the use or non-use of adhesion promoting substances.

Studies that did not discuss yttria-stabilized zirconia, book chapters, and case reports were excluded. Additionally, in vitro investigations with a sample size per treatment of less than 8 specimens were discarded (although they were used in discussion) as ISO 29022:2013 and ISO 11405:2015 recommend at least 15 valid bond strength measurements per substrate/adhesive combination, and using eight specimens typically provides this minimum when multiple bonded areas are prepared per sample [41,43]. Assays that did not include comparisons with untreated zirconia surfaces, assays that did not subject the material to shear forces and finally studies of adhesion to orthodontic brackets and intraradicular posts were not included.

During the reading of complete articles, including literature reviews and meta-analyses, a snowball process was performed finding additional articles to the database search (Figure 1). Reference lists of selected articles were reviewed. Discrepancies between reviewers were resolved through discussion and consensus. These procedures were implemented to minimize bias and ensure methodological rigor. The authors declare that during the process of conducting the systemic literature review, the PRISMA (Preferred Reporting Items for Systematic Reviews and Meta-Analyses) guidelines were followed. We did not have any additional registration information. So, all included studies were evaluated following the PRISMA checklist (Supplementary Table S1). This checklist was designed to improve transparency and quality of systematic reviews and meta-analyses, ensuring compliance with established inclusion and exclusion criteria (Figure 1).

The results were transferred to an Excel (v.365, Microsoft Corporation, Redmond, WA, USA) spreadsheet to conduct an exploratory grouped analysis based on the selected articles, without discriminating against the materials used. This approach allowed for a clearer observation of potential differences between the use of no coating, a silica coating alone, and a silica coating combined with an adhesion promoter.

## 3. Results

**Figure 2:**
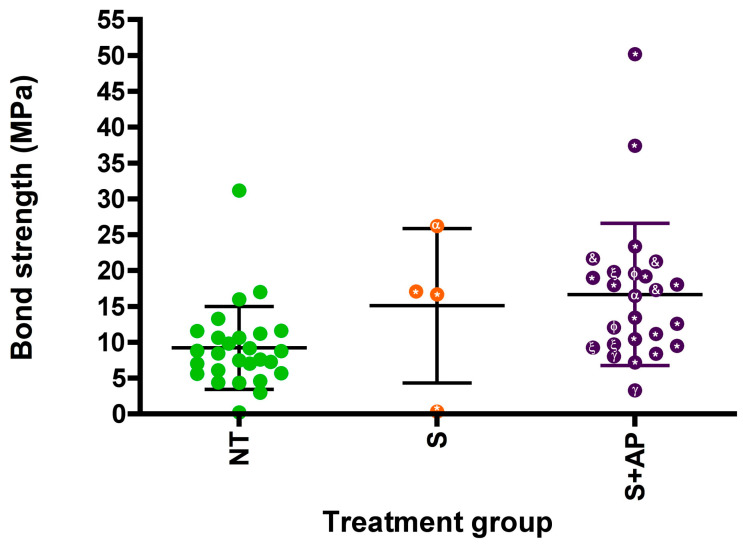
Distribution of adhesive bond strength (MPa) according to the general type of silica surface treatment. Exploratory scatter plot showing the distribution of adhesive bond strength values (in MPa) reported in studies grouped by treatment strategy: no treatment (NT), silica-based treatment only (S), and silica-based treatment combined with an adhesion promoter (S + AP). Each point represents individual data extracted from the studies included. Data points are coded according to the type of silica treatment: tribochemical coating (*), sputtering (ξ), atomic layer deposition (ALD (α)), sol–gel (γ), immersion coating (&), and other silica-based coatings (ϕ). Bars represent the mean ± standard deviation for each treatment group. Although not statistically comparable due to differences in methodology among studies, the plot suggests a clear trend of increasing bond strength with the incorporation of silica treatments, particularly when combined with adhesion promoters.

This review included 27 studies conducted between 2010 and 2024, which addressed different zirconia surface treatments to improve SACR adhesion. The analysis of these studies, summarized in Table 1, reveals significant findings for clinical practice, considering the reported techniques for generating silica coating on the zirconia surface. Five main techniques were identified for generating silica coating on zirconia: atomic layer deposition (ALD) (n = 2 articles), sol–gel (n = 3 articles), plasma-assisted sputtering (n = 3 articles), dip coating (n = 2 articles), and most frequently, tribochemical silica coating using systems such as Rocatec (3M ESPE, St Paul, MN, USA) or Cojet (3M ESPE, St Paul, MN, USA) (n = 17). The studies demonstrate that silica incorporation, regardless of the technique used, improves shear bond strength compared to untreated zirconia, as detailed in Table 1 and Figure 2. In some cases, this improvement even exceeds that obtained with aluminum oxide sandblasting or laser treatments, highlighting the potential of these techniques to optimize SACR adhesion [27,50,51,52].

The data consistently demonstrate that surface treatment is fundamental, highlighting aluminum oxide sandblasting as an effective method, with increases in adhesive bond strength at the zirconia–cement interface ranging from 50% to 300% compared to untreated surfaces [53,55,61,66]. The combination of tribochemical silica treatment (TBS) plus the use of a coupling agent showed the most favorable values, with shear bond strength values reaching up to 50.21 MPa in Lin et al.’s study [50] and 37.4 MPa in Da Silva et al.’s study [58], representing some of the highest measurements across all studies.

The highest shear bond strength is not only related to the use of different physicochemical and/or mechanical treatments of the zirconia surface but also depends on the type of resin cement and the use of chemical adhesion promoting agents (n = 21 articles). In this review, it was found that studies using resin cements with incorporation of functional monomers such as the 10-methacryloxydecyl dihydrogen phosphate (MDP) molecule within their composition, such as Panavia F 2.0 and Clearfil SA (both from Kuraray Noritake Dental Inc., Tokyo, Japan), generally showed superior performance, especially when used in conjunction with specific adhesion promoters such as Clearfil Ceramic Primer (Kuraray Noritake Dental Inc., Tokyo, Japan) or RelyX Ceramic Primer (3M ESPE, St. Paul, MN, USA) [57,58,67]. Other resin cements such as RelyX U100/U200/Ultimate (3M ESPE, St. Paul, MN, USA) showed consistent results across multiple studies. Multilink resin cement (Ivoclar Vivadent, Schaan, Liechtenstein) also demonstrated good performance, particularly when used with adhesion promoters.

An evolution in adhesion techniques is observed in the most recent studies (2020–2023), evidencing a trend toward combined treatments and systematic use of primers. This suggests that the most effective protocol consists of physicochemical and/or mechanical treatment followed by application of an MDP-containing primer [52,69,70]. Among these MDP-containing adhesion promoters, Clearfil Ceramic Primer (Kuraray Noritake Dental Inc., Tokyo, Japan) and RelyX Ceramic Primer (3M ESPE, St. Paul, MN, USA) stand out.

In several analyzed studies, silica surface treatments on zirconia, especially when combined with adhesion promoters, showed significant improvements in adhesive strength compared to the untreated group. For example, in Su et al.’s study [23], silica particle treatment increased adhesion by 182%, while the combination of silica and adhesion promoter elevated it by 389%. Similarly, Karami Zarandi et al. [66] reported improvements of up to 104% when using aluminosilicate-based treatments. Other studies such as those by Peçanha [68] and Bitencourt [68] also confirmed relevant increases in adhesion, with improvements of 167% and 65%, respectively.

On the other hand, thermal aging was considered within the experimental groups in 16 articles. The ISO 11405:2015 standard suggests thermal aging by thermocycling with temperature changes between 5 °C and 55 °C [43]. Although a specific number of cycles is not established, this standard recommends 500 cycles. In the reviewed literature, at least 5000 cycles were performed in the experimental groups. In general, all experimental groups, with different surface treatments and adhesion of different resin cements, showed a decrease in adhesive bond strength values at the zirconia–cement interface after thermocycling; that is, simulating extreme temperature changes in the mouth. The reduction in strength was lower in silica-treated groups (approximately 20% reduction) compared to control groups without silica (approximately 24% reduction).

The primary method for identifying failure type in the review studies was stereomicroscope observation. In treatments with silica coating on the zirconia surface, cohesive or mixed failures were mostly observed. In contrast, in untreated zirconia or with aluminum oxide sandblasting, failures were mostly adhesive or mixed.

## 4. Discussion

The results obtained in this systematic review demonstrate that silica particle surface treatments applied to yttria-stabilized zirconia (3Y-TZP) significantly improve the bond strength of resin-based luting agents under shear forces. This effect is evident in both specimens subjected to thermal aging and those without thermocycling, suggesting more stable and durable adhesion with potential clinical benefits [50,54,57]. Five main methods for silica coating of zirconia were identified: tribochemical silica coating, cathodic sputtering, atomic layer deposition (ALD), sol–gel technique, and dip coating. Although these methods share the goal of improving chemical affinity between zirconia and adhesive systems, their efficacy varies considerably due to differences in application technology, control of coating thickness, and chemical stability of the formed layer.

Tribochemical surface treatment (TBS) consists of creating a thin silica layer on the zirconia surface through a physicochemical process, in which aluminum oxide particles coated with silica are embedded onto zirconia through pressurized sandblasting [51,60]. The kinetic energy generated during this process enables incorporation of the particles, forming a silicatized surface. This silica layer on the zirconia surface not only increases the surface roughness but also introduces silanol groups that enhance chemical interaction with adhesive systems, thus facilitating adhesion of resin-based luting agents [49,61].

The data analyzed in this review suggest that combining TBS of zirconia with a primer and resin cement containing the functional monomer MDP in their composition represents one of the most effective strategies for optimizing adhesion of resin-based luting agents to zirconia. This trend is consistently observed across multiple studies, highlighting superior resistance values, such as those reported by Lin et al. [50], Shin [58], and Peçanha [68], where a 65% increase in adhesion was observed compared to untreated surfaces and 25% compared to surfaces treated only with aluminum oxide sandblasting.

Despite the demonstrated effectiveness of tribochemical treatment combined with MDP-containing primers and cements, methodological variability among the included studies must be considered. Application protocols differed in aspects such as particle size (CoJet: 30 μm vs. Rocatec: 110 μm), application time (between 10 and 20 s), and pressure, all of which may affect the uniformity and efficacy of the silica layer on zirconia [49,50,58,71]. Additionally, the type of primer used and its chemical compatibility with the cement represent critical factors influencing adhesion [40]. This heterogeneity limits the establishment of a universal protocol but allows the identification of consistent trends that guide clinical practice.

However, despite the proven effectiveness of tribochemical treatment, some studies have reported certain limitations. Possible adverse effects associated with its application have been described, particularly induction of microfractures on the zirconia surface due to tetragonal to monoclinic phase transformation, a phenomenon that compromises the structural integrity of the material [72,73]. There is also a significant variability in depth and uniformity of silica particle penetration, influenced by factors such as working pressure (commonly between 2.5 and 2.8 bar), particle size, application time, and nozzle distance [74,75,76,77]. Increases in the first three parameters may heighten the risk of surface damage, including cracks, mass loss, or crystalline changes, which could reduce the long-term stability of the zirconia–cement interface [76,77].

Sputtering and atomic layer deposition (ALD) differ mainly in their deposition mechanism and level of control. Sputtering is a physical technique involving cathodic pulverization of a solid target through ionic bombardment, whereas ALD is a sequential chemical process that deposits atomic layers in a controlled manner onto the substrate surface [78,79]. Both techniques produce highly uniform, homogeneous silica films firmly adhered to the zirconia surface, allowing more predictable and stable coatings compared to traditional methods such as TBS [17,40,80]. Moreover, unlike TBS, these techniques have not shown evidence on inducing structural changes in zirconia that could compromise its mechanical properties [17,18,19].

Nevertheless, the main challenge of both techniques lies in the requirement for specialized equipment: high-precision vacuum chambers, plasma sources, or chemical deposition reactors, which are expensive and not available in standard clinical settings. Among them, ALD demands more expensive and sophisticated equipment than cathodic sputtering, due to its complex design, higher level of control, and automation. However, ongoing technological advancements and potential industrial-scale development may enhance the accessibility of these surface treatments in the near future [17,19,42].

In studies such as Yan [17], significantly higher bond strength values were reported after thermocycling with the ALD technique, reaching up to 16.49 MPa, compared to values below 10 MPa observed in conventional treatments such as aluminum oxide sandblasting. Similarly, El-Shrkawy [27] reported bond strengths of up to 17.8 MPa using cathodic sputtering as a single treatment. These improvements in adhesion at the zirconia—cement interface are attributed to the formation of a nanometric silica layer with controlled thickness and excellent adherence, which enhance interaction with adhesion-promoting agents and, consequently, with resin cements [17,27,34]. These characteristics contribute to more predictable and stable long-term adhesion.

The sol–gel method, although less studied (n = 3), has demonstrated promising results, particularly in the study by Campos [62]. This technique allows formation of a silica-based film on the substrate through immersion, spraying, or spin-coating. The thickness of this film depends on a combination of variables such as sol concentration, immersion or rotation speed, drying time, and environmental conditions, all of which can influence the outcome [81]. Additionally, the chemical reactions of hydrolysis and condensation involved may not follow linear progression. During the drying stage, uneven shrinkage of the gel can occur, potentially leading to crack formation and thickness irregularities [22,82].

Nevertheless, this technique enables the formation of a chemically stable coating on zirconia through the establishment of covalent bonds between silanol groups of the coating and hydroxyl groups present on the substrate surface [66,83]. The relative lack of coating uniformity obtained with this technique could explain the variability observed in adhesive strength values, which in some studies range from 3.3 MPa to 11.6 MPa. According to Matinlinna et al. [34], the absence of precise control over reaction parameters during the sol–gel process can lead to coatings with irregular thicknesses and variable porosity, which compromises formation of stable chemical bonds between the silica layer and the silane coupling agent [34]. These surface irregularities may also impair adhesive penetration and compromise polymerization at the interface [3]. Although the sol–gel method is a relatively simple and low-cost technique compared to techniques such as ALD or sputtering, its lack of deposition control can negatively affect coating quality, potentially resulting in less predictable adhesion and clinical challenges related to the long-term durability of adhesive systems.

Dip coating was evaluated in two studies included in this review, showing variable results in terms of adhesive strength, with values ranging between 12.27 MPa and 21.28 MPa [23,66]. This variability can be mainly attributed to differences in application protocols that were not fully detailed in the methodology, particularly regarding immersion time, precursor sol concentration, substrate withdrawal speed, and drying conditions [84,85]. Furthermore, the use of different resin cement and adhesion promoters from different commercial houses in both studies introduce additional variables that limit direct comparison of results.

Although this technique is accessible and can be performed at room temperature, facilitating its implementation in clinical or laboratory environments, its main limitation lies in low predictability of coating thickness and uniformity [84,86]. These inconsistencies directly affect adhesive interface quality and, therefore, bond strength values. Furthermore, lack of precise control over reaction parameters can generate coatings with irregular thicknesses, which compromises formation of stable chemical bonds between the silica layer and resin cement [34,82]. Regarding the evaluated dependent variables, the two analyzed studies reported significant improvement in adhesive strength compared to untreated zirconia (increases of 180–250%), along with favorable change in failure type and a higher prevalence of mixed failures compared to controls. This suggests a more effective and stable adhesion [23,66].

The combination of physical/mechanical surface treatments with silica and the application of chemical adhesion-promoting agents appears to be a determining factor to maximize bond strength to zirconia [87,88]. Results from this review show that 21 of the included studies incorporated adhesion promoters, evidencing significant synergistic effects when combined with surface treatments. Particularly, adhesion promoters containing the functional monomer MDP consistently demonstrated superior shear bond strength at the zirconia- resin cement interface, with increases up to 60–80% compared to other promoters lacking MDP [27,49,62]. This enhanced performance is attributed to the dual functionality of this molecule: its phosphate groups form stable chemical bonds with zirconia metal oxides (Zr-O-P), while its methacrylate groups copolymerize with the organic matrix of resin cement, strengthening cohesion of the cementing system [89,90,91]. This chemical interaction complements the increased surface area and physicochemical modifications provided by silica-based surface treatments. The highest bond strength values (exceeding 15 MPa before thermocycling) were consistently observed in protocols combining silica coatings with subsequent application MDP-containing primers or resin cements [57,59]. These findings support the notion that achieving durable adhesion to zirconia requires a dual approach: structural surface modification through silicatization and chemical interaction mediated by functional monomers designed to bond with the modified surface.

The thermocycling process, which simulates intraoral thermal aging through repeated cycles of exposure to extreme temperatures, allows evaluation of long-term stability of adhesion at the zirconia-cement interface with different treatment protocols. As expected, all experimental groups included in this review exhibited a reduction in bond bond strength following thermocycling. However, surface modifications using advanced techniques such as atomic layer deposition (ALD) and sputtering were able to preserve bond strength values above 10 MPa even after 10,000 thermal cycles, substantially higher than the approximately 3 MPa observed in untreated zirconia under the same conditions [17,27]. These findings suggest that silica-based coatings contribute to a more stable adhesive interface, capable of withstanding cyclic thermal stress typical of the oral environment. This improved stability is likely due to the formation of durable chemical bonds among the silica layer, silane coupling agents, and the resin cement, which exhibit superior hydrolytic resistance compared to other adhesion mechanisms [42,87,88].

The analysis of failure modes following mechanical testing provides valuable insights into the quality and integrity of the adhesive interface. In groups subjected to silica-based surface treatments, cohesive and mixed failures were predominant, suggesting that bond strength of the zirconia-cement interface exceeded cohesive strength of resin cement or the silica layer itself [43,47,48]. This failure pattern contrasts notably with that observed in control groups and those treated only with aluminum oxide sandblasting, where adhesive failures prevailed [44,51,53]. This finding reinforces the hypothesis that silica treatments not only increase initial bond strength but also substantially modify the nature of the adhesive interface, facilitating a more effective mechanical and chemical integration between the resin cement and the zirconia substrate.

One of the most relevant limitations identified in this review is the lack of standardization in experimental protocols between different studies, and even within individual studies [42,58]. This methodological heterogeneity is evident in several key aspects: (1) differences in coating application parameters, including exposure time, working pressure, working distances, substrates types, and particle sizes; (2) variations in pre- and post-treatment cleaning procedures, ranging from specialized zirconia surface cleaners to alcohols, ultrasonic baths, and/or pressurized water jets, which can alter surface energy and wettability; (3) differences in resin cements employed, each with distinct chemical compositions and rheological properties; (4) inconsistent use of adhesion promoters, with or without MDP; and (5) variability in thermocycling protocols, including number of cycles and temperature ranges, with some studies omitting this aging procedure entirely. Another important limitation identified in this review was the restriction to studies that included untreated zirconia as a control group and used shear bond strength (SBS) as the testing method. Including a common baseline (untreated zirconia) allowed a clearer assessment of the relative effectiveness of each surface treatment, as reported in the analyzed articles.

These methodological discrepancies complicate direct comparisons among studies and hinder the ability to draw definitive conclusions about optimal clinical protocols. Moreover, they reduce the internal validity of individual experiments and may partially account for the variability in reported outcomes. While the overall trend toward improved bond strength with silica-based surface treatments remains consistent, these limitations underscore the need for standardized experimental designs and reporting criteria. Future studies should aim to reduce these sources of variability to enhance the robustness and translatability of their findings into clinical practice.

The clinical implications of these findings are significant for dentists, institutions providing dental services, dental laboratory technicians, as well as patients and the healthcare system in general. Improvement in adhesive bond strength at the zirconia-cement interface and its stability over time could translate into substantial reduction of clinical complications such as prosthetic dislodgments, marginal microleakage, secondary caries, and fracture of remaining tooth structure, which would imply additional costs for patients [9]. These complications not only compromise treatment success but also generate additional costs for patients and public health systems in general. For clinical professionals, implementation of silica-based treatments represents an opportunity to simplify adhesive protocols, increase result predictability, and improve restoration longevity. For laboratory technicians, these findings support the incorporation of surface treatment technologies as an integral part of zirconia restoration fabrication process, which could improve quality and longevity of delivered restorations. Finally, for the dental materials industry, these findings open the door to the development of more efficient and standardized solutions that respond to current clinical needs, with potentially positive impact on global healthcare system sustainability.

However, it is important to note that most silica coating methods, except tribochemical coating, require specialized equipment not routinely available in clinical environments or even in many dental laboratories. Techniques such as ALD and cathodic sputtering require vacuum chambers, plasma sources, or high-precision chemical reactors whose cost can range between $100,000 and $500,000 USD, depending on system complexity and automation capacity. This level of investment limits immediate applicability in daily dental practice, restricting it mainly to advanced research centers or laboratories with more robust technological infrastructure. Nevertheless, progressive introduction of these technologies in the biomedical field represents a promising field for the future. Techniques such as atomic layer deposition and plasma-assisted coating offer nanometric control over coating thickness and uniformity, which could translate into more stable, durable, and predictable zirconia-cement interfaces [92,93]. As these technologies become more accessible and simplified clinical versions are developed, their implementation could transform adhesive protocols in restorative dentistry.

Collectively, findings from this systematic review confirm that silica surface treatments, especially when combined with MDP-containing systems, represent an effective strategy for improving adhesion to zirconia. While methodological differences exist between studies, the general trend is clear: silica surface modification improves both bond strength and long-term stability of the zirconia-cement interface. Nevertheless, this review has some limitations, including the exclusive inclusion of in vitro studies, which may not fully replicate intraoral conditions; variability in study designs, coating parameters, and thermocycling protocols; and the absence of standardized aging conditions across all studies. These factors may influence the generalizability of the findings to clinical practice.

Future research should focus on conducting well-designed in vitro studies with standardized protocols, complemented by in situ or clinical studies that validate laboratory findings under real oral conditions. Comparative studies evaluating emerging coating technologies against more conventional treatments are also recommended to establish evidence-based, accessible, and reproducible clinical protocols.

Based on the findings of this review, future studies are encouraged to standardize zirconia surface treatment protocols, including coating methods, application parameters, surface cleaning procedures, and the use of adhesion promoters, to improve consistency and facilitate meaningful comparisons across investigations. From a clinical perspective, the combination of silica-based surface treatments with adhesion promoters containing functional monomers such as MDP has demonstrated superior bond strength and improved interfacial stability and should therefore be prioritized in adhesive protocols.

Furthermore, it is advisable to promote the development and adaptation of advanced coating technologies, such as atomic layer deposition (ALD) and sputtering, to make them more accessible and practical for clinical or dental laboratory settings. The systematic inclusion of failure mode analysis is also recommended, as it provides essential qualitative information about the nature and integrity of the adhesive interface—complementing quantitative bond strength measurements. Finally, standardized thermocycling protocols should be incorporated into all future studies to better simulate intraoral conditions and assess long-term performance.

## 5. Conclusions

Silica-based surface treatments have proven to be an effective strategy for enhancing the adhesion of resin cements to zirconia. The available evidence suggests benefits not only in initial bond strength but also in long-term stability, even after thermal aging. Notably, the combination of silica coatings with adhesion promoters containing functional monomers such as MDP has shown up to an 80% increase in bond strength compared to treatments without MDP. Furthermore, the studies included in this review reported a predominance of cohesive and mixed failures in the groups treated with silica, indicating a stronger and more reliable adhesive interface. These findings underscore the importance of employing combined protocols that involve physicochemical surface modifications (such as tribochemical silica coating) along with the use of adhesion promoters and MDP containing resin cements.

## Figures and Tables

**Table 1:** Selected articles for the review and results in MPa under shear stress. Treatments, highest resistance values in MPa obtained in each study for different treatments without thermocycling, resin cements used in tests, and use of adhesion promoters are shown.

	Experimental Group Results (MPa)						Resin Cement Used in the Study	Adhesive Promoter
Author/Year	Control Group (No Treatment/APA)		Other Surface Treatments		Silica Coating			
Akyil [53]	NT + AP APA + AP	17.02 23.46	LS Er:YAG + AP, LS Nd:YAG + AP, LS CO2 + AP, APA+ LS Er:YAG + AP, APA + LS Nd:YAG + AP, APA + LS CO2 +AP,	19.69 15.62 22.35 14.85 20.82 19.30	TBS + AP	23.39	Clearfil Esthetic (Kuraray, Okayama, Japan)	Clearfil Ceramic Primer (Kuraray, Okayama, Japan)
Lin [50]	NT	16.7			TBS TBS + AP	31.17 50.21	Maxcem (Kerr, Orange, CA, USA) Smartcem (Dentsply, York, PA, USA) Rely X Unicem (3M ESPE, Seefeld, Germany) Breeze (Pentron clinical, Orange, CA, USA) Biscem (Bisco, Schaumburg, IL, USA) Set (SDI, Bayswater, VIC, Australia) Clearfil SA luting (Kuraray, Okayama, Japan)	Clearfil ceramic primer (Kuraray, Okayama, Japan)
Subaşı [54]	NT APA	0.22 0.74	LS Er:YAG APA + LS Er:YAG	0.34 0.75	TBS	0.35	Relyx U100 (3M ESPE, Seefeld, Germany) Clearfil Esthetic (Kuraray, Okayama, Japan)	No
Queiroz [42]	NT NT + AP APA + AP	3.1 5.7 16.3			PC + AP	9.7	Multilink (Ivoclar-Vivadent, Schaan, Liechtenstein) Panavia F (Kuraray, Okayama, Japan) Relyx U100 (3M ESPE, Seefeld, Germany)	Monobons S (Ivoclar Vivadent, Schaan, Liechtenstein) Metal/zirconia primer (Ivoclar Vivadent, Schaan, Liechtenstein)
Liu [55]	NT + AP APA + AP	8.8 19.7	VC ZPC	15.9 14. 7	SCD + AP	19.2	RelyX Unicem (3M ESPE, Seefeld, Germany)	RelyX Ceramic Primer (3M ESPE, Seefeld, Germany)
Gomes [56]	NT	7.5	LS Er:YAG	5.7	TBS + AP LS Er:YAG + TBS + AP	17.3 18.9	BiFix SE (Voco, Cuxhaven, Germany) Clearfil SA (Kuraray, Okayama, Japan)	RelyX Ceramic Primer (3M ESPE, Seefeld, Germany)
Román-Rodriguez [57]	PA + AP	14.9	VC	27.21	TBS + AP	13.44	Panavia F2.0 (Kuraray, Okayama, Japan) Panavia V5 (Kuraray, Okayama, Japan)	No
Erdem [44]	NT APA	3.0 14.8	LS Er:YAG	5.0	TBS + AP	19.8	Panavia F 2.0 (Kuraray, Okayama, Japan) Relyx U 100 (3M ESPE, Seefeld, Germany) Clearfill Esthetic (Kuraray, Okayama, Japan) Super-bond C&B (Sun Medical Co. Moriyama, Shiga, Japan) Multilink Automix (Ivovlar-Vivadent, Schaan, Liechtenstein)	No
Shin [58]	NT APA NT + AP APA + AP	4.11 6.98 8.47 14.12			TBS + AP	7.21	Panavia F 2.0 (Kuraray, Okayama, Japan) Clearfil SA (Kuraray, Okayama, Japan)	Z-Prime Plus (Bisco, Schaumburg, IL, USA) Sil (3M ESPE, Seefeld, Germany)
Da Silva [59]	NT AP	16.0 36.2			TBS + AP	37.4	RelyX ARC (3M ESPE, Seefeld, Germany) Relyx Unicem (3M ESPE, Seefeld, Germany)	Alloy primer (Kuraray, Okayama, Japan) Espe Sil (3M ESPE, Seefeld, Germany)
Yenisey [60]	NT AP APA APA + AP	4.62 5.55 4.89 7.26	APA + LS Er:YAG + AP	6.70	APA + TBS + AP	11.19	Panavia F2.0 (Kuraray, Okayama, Japan)	No
Re [61]	NT APA	13.29 16.24			TBS	17.1	Clearfil SA (Kuraray, Okayama, Japan)	No
Mahmoodi [51]	NT APA		LS Nd:YAG		TBS + AP		Panavia F2.0 (Kuraray, Okayama, Japan) Clearfil SA (Kuraray, Okayama, Japan)	Monobond S (Ivoclar Vivadent, Schaan, Liechtenstein)
Campos [62]	NT + AP	11.64			SG + AP	3.31	Variolink II (Ivoclar Vivadent, Schaan, Liechtenstein)	Monobond Plus (Ivoclar Vivadent, Schaan, Liechtenstein) Monobond S (Ivoclar Vivadent, Schaan, Liechtenstein)
El-Shrkawy [27]	NT + AP APA + AP	6.1 13.2	PT + AP	17.8	PC + AP	19.6	Multilink (3M ESPE, Seefeld, Germany)	Metal/zirconia primer (not specified)
Zanatta [63]	NT APA	7.28 13.31	LS Nd:YAG	20.99	TBS + AP	18.05	RelyX U200 (3M ESPE) Bifix SE (Voco, Cuxhaven, Germany)	Ceramic Bond (Voco, Cuxhaven, Germany)
Vicente Prieto [64]	NT APA	4.4 3.6	LS Femtosecond laser	10.8	TBS + AP	9.5	Clearfil SA (Kuraray, Okayama, Japan)	RelyX deramic primer (3M ESPE, Seefeld, Germany)
Skienhe [65]	NT + AP APA + AP	11.58 17.59			SCD + AP	12.07	RelyX Ultimate (3M ESPE, Seefeld, Germany)	Scotchbond Universal (3M ESPE, Seefeld, Germany)
Zhou [52]	NT + AP APA + AP	8.83 10.74	LS Femtosecond laser + AP	13.76	PC + AP APA + PC + AP LS + PC + AP	9.26 9.84 13.51	Multilink N (Ivoclar Vivadent, Schaan, Liechtenstein)	Monobond N (Ivoclar Vivadent, Schaan, Liechtenstein)
Karami Zarandi [66]	NT + AP APA + AP	10.63 18.25			IC (silica) + AP IC (aluminosilicate) +AP	17.98 21.66	Clearfil SA (Kuraray, Okayama, Japan)	Clearfil SE (Kuraray, Okayama, Japan) Clearfil porcelain bond activator (Kuraray, Okayama, Japan)
Su [23]	NT APA + AP	4.35 12.27			IC + AP	21.28	RelyX Ultimate (3M ESPE, Seefeld, Germany)	Monobond N (Ivovlar Vivadent, Schaan, Liechtenstein) Z-Prime (Bisco, Schaumburg, IL, USA)
Yan [18]	NT + AP	7.05			SG + AP SCD + AP ALD + AP	8.07 8.58 16.49	Choice 2 (Bisco, Schaumburg, IL, USA)	Bis-Silane (Bisco, Schaumburg, IL, USA)
Souza-Filho [67]	NT + AP	5.6	LS Er:YAG 1 + AP LS Er:YAG 2 + AP LS Er:YAG 3 + AP	5.7 4.6 4.3	TBS + AP	8.4	PanaviaF 2.0 (Kuraray, Okayama, Japan) RelyX U200 (3M ESPE, Seefeld, Germany)	RelyX ceramic Primer (3M ESPE, Seefeld, Germany)
de Figueiredo [49]	NT + AP	9.2	OT (fluorine nanofilm) + AP	11.1	TBS + AP SCD + AP	19.0 3.6	Panavia F (Kuraray, Okayama, Japan)	Clearfil SE (Kuraray, Okayama, Japan)
Peçanha [68]	NT APA APA + AP	7.61 10.1 9.56			TBS + AP TBS + AP(MDP)	12.6 10.4	Panavia F 2.0 (Kuraray, Okayama, Japan) RelyX U200 (3M ESPE, Seefeld, Germany)	RelyX ceramic Primer (3M ESPE, Seefeld, Germany) Alloy Primer (Kuraray, Okayama, Japan)
Bitencourt [69]	NT APA	9.83 26.75			ALD	26.23	Multilink (Ivoclar Vivadent, Schaan, Liechtenstein)	No
Sarıkaya [70]	NT + AP APA + AP	11.2 11.28	OT (hot acid solution) + AP OT (Aluminum Nitrite) + AP	18.06 8.79	TBS + AP	10.43	Panavia F 2.0 (Kuraray, Okayama, Japan) RelyX Unicem (3M ESPE, Seefeld, Germany)	Clearfill ceramic primer (Kuraray, Okayama, Japan)

NT: no treatment. APA: aluminum oxide sandblasting. LS: laser. VC: vitreous ceramic. ZPC: zirconia particle coating. PT: plasma. OT: other treatment (specified in each article). Silica coatings by means of TBS: tribochemical. SG: sol–gel technique. IC: immersion. ALD: atomic layer deposition. PC: sputtering. AP: adhesion promoter. SCD: other silica coating method.

## Data Availability

No new data were created or analyzed in this study. Data sharing is not applicable to this article.

## Supplementary Materials

The following supporting information can be downloaded at https://www.mdpi.com/article/10.3390/dj13090426/s1. Table S1: PRISMA 2020 Checklist completed for the present systematic review, indicating the page number or section where each item is addressed.

## Appendix A

**Table A1:** Search terms and algorithms applied across various databases.

Database	Search Algorithm	Applied Limits
Scopus	(TITLE-ABS-KEY (zirconi*) OR TITLE-ABS-KEY (“yttria stabilized zirconia”) OR TITLE-ABS-KEY (“3y tzp”) AND TITLE-ABS-KEY (“surface treatment*”) OR TITLE-ABS-KEY (“thin film*”) OR TITLE-ABS-KEY (coating*) OR TITLE-ABS-KEY (“surface conditioning”) AND TITLE-ABS-KEY (“silicon dioxide”) OR TITLE-ABS-KEY (silica) AND TITLE-ABS-KEY (“resin cement*”) OR TITLE-ABS-KEY (“dual cure resin cement”) OR TITLE-ABS-KEY (“luting cement*”) AND TITLE-ABS-KEY (“bond strength”) OR TITLE-ABS-KEY (“bond strength test” ) AND NOT TITLE-ABS-KEY (implant))	None
Web of Science	ALL = (Zirconi* (Topic) or “yttria stabilized zirconia” (Topic) and “surface treatment*” (Topic) or “surface conditioning” (Topic) and “silicon dioxide” (Topic) or silica (Topic) and “resin cement*” (Topic) or “dual cure resin cement” (Topic) and “bond strength” (Topic) not “dental implant*” (Topic) not “Human Dentin” or Dentin (Title) and Preprint Citation Index (Exclude—Database))	Principal collection of Web of Science
PubMed	((((((((“zirconi*”[Title/Abstract] OR “yttria stabilized zirconia”[Title/Abstract]) AND “surface treatment*”[Title/Abstract]) OR “thin film*”[Title/Abstract] OR “surface conditioning”[Title/Abstract]) AND “silicon dioxide”[Title/Abstract]) OR “silica”[Title/Abstract]) AND “resin cement*”[Title/Abstract]) OR “dual cure resin cement”[Title/Abstract]) AND “bond strength”[Title/Abstract]) NOT “implant”[Title/Abstract]	none
ScienceDirect	(zirconia OR “yttria stabilized zirconia”) AND (“surface treatment” OR “thin film”) AND (“silica” OR “Silicon dioxide”) AND (“bond strength”) AND NOT (dental implant))	none
EBSCO	TI zirconia OR TI yttria-stabilized zirconia AND surface treatment AND bond strength AND silicon dioxide AND resin cement	Academic Journals Thesaurus term bond strengths
